# Special Issue “Arbovirus Diagnostics”

**DOI:** 10.3390/v16081182

**Published:** 2024-07-24

**Authors:** Claudia Fortuna, Giulia Marsili, Giulietta Venturi

**Affiliations:** National Reference Laboratory for Arboviruses, Department of Infectious Diseases, Istituto Superiore di Sanità, Viale Regina Elena 299, 00161 Rome, Italy; gulia.marsili@iss.it (G.M.); giulietta.venturi@iss.it (G.V.)

## 1. Introduction

Arboviruses are pathogens transmitted mainly by mosquitoes, ticks, and sandflies. Their ability to cause significant human disease highlights the critical importance of diagnostic strategies to mitigate their impact on public health. The choice of diagnostic methods varies widely depending on the virus and the stage of infection, but the use of molecular techniques, where possible, remains the best tool for accurate and specific diagnosis. The advantages and disadvantages of different diagnostic methods determine their applicability in diagnosing arbovirus infections [1]. In particular, surveillance efforts for some arboviruses such as dengue (DENV), Zika (ZIKV), and chikungunya (CHIKV) viruses have relied heavily on molecular assays due to their accuracy in identifying viral RNA in clinical specimens.

## 2. Overview of the Published Articles

The articles published in the Special Issue “Arbovirus Diagnostics” significantly contribute to the enhancement and optimization of diagnostic methodology. A study of molecular and serological testing on different groups during the CHIKV epidemic in Myanmar highlighted the need for comprehensive diagnostics [2]. In Vietnam, where CHIKV has been historically understudied, recent serological and genomic studies have revealed high prevalence rates and the presence of specific viral genotypes, emphasizing the importance of robust surveillance programs to monitor disease impact and coordinate public health interventions [3]. Despite progress, the need for continuous evaluation and the refinement of diagnostic protocols is still critical in a global scenario where multiple arboviruses are circulating in the same areas, with overlapping clinical presentation, making differential diagnosis an issue [4]. Studies have explored innovative approaches using non-invasive samples such as oral fluids and urine for CHIKV detection via RT-qPCR, offering potential alternatives to invasive methods [5]. In the Mediterranean, Toscana virus (TOSV) presents diagnostic challenges due to overlapping symptoms with other neuro-invasive viruses. PCR testing of biological fluids such as cerebrospinal fluid and urine is critical. Optimizing diagnostic protocols, such as diluting urine to enhance sensitivity, enhances the reliability of RT-PCR-based diagnostics for TOSV and potentially other arboviruses [6]. Research into nanoluciferase-expressing ZIKV variants shows promise for vaccine development and therapeutic interventions, although current options remain investigational [7]. The development of a specific real-time PCR method enhances detection sensitivity for Phleboviruses in sandflies and vertebrate hosts, improving our understanding of virus epidemiology and guiding targeted control measures [8]. The global emergence of West Nile virus highlights the need for advanced genomic surveillance methods. Novel whole-genome amplicon-based sequencing approaches provide effective coverage across hosts and insights into virus evolution and transmission [9].

## 3. Conclusions

The diagnosis of arboviral infections is crucial because timely and accurate identification can guide clinical management and treatment, reducing morbidity and mortality; it also helps in implementing public health measures to control and prevent outbreaks and improving our understanding of the epidemiology of these infections. Continued research and innovation of diagnostic technologies will be pivotal in mitigating the impact of these complex pathogens on global health.
